# Protein arginine methylation: an emerging regulator of the cell cycle

**DOI:** 10.1186/s13008-018-0036-2

**Published:** 2018-03-20

**Authors:** Anita E. Raposo, Sabine C. Piller

**Affiliations:** School of Science and Health, Western Sydney University, Penrith, NSW 2751 Australia

**Keywords:** Protein arginine methylation, Cell cycle regulation, p53, Cancer, DNA repair

## Abstract

Protein arginine methylation is a common post-translational modification where a methyl group is added onto arginine residues of a protein to alter detection by its binding partners or regulate its activity. It is known to be involved in many biological processes, such as regulation of signal transduction, transcription, facilitation of protein–protein interactions, RNA splicing and transport. The enzymes responsible for arginine methylation, protein arginine methyltransferases (PRMTs), have been shown to methylate or associate with important regulatory proteins of the cell cycle and DNA damage repair pathways, such as cyclin D1, p53, p21 and the retinoblastoma protein. Overexpression of PRMTs resulting in aberrant methylation patterns in cancers often correlates with poor recovery prognosis. This indicates that protein arginine methylation is also an important regulator of the cell cycle, and consequently a target for cancer regulation. The effect of protein arginine methylation on the cell cycle and how this emerging key player of cell cycle regulation may be used in therapeutic strategies for cancer are the focus of this review.

## Background

The cell cycle allows cells to divide and is characterised by the replication of DNA and the subsequent division of duplicated chromosomes into two daughter cells [1]. Regulatory proteins including the tumor suppressor protein p53 and the retinoblastoma protein (pRb) monitor the cell cycle and initiate pathways in response to DNA damage [2]. Cancer is considered to be a disease of the cell cycle [3] and as such these cell cycle regulatory proteins are often found to be deregulated in cancer. Protein arginine methylation is a post-translational modification often upregulated in cancer and other diseases [4], contributing to the deregulation of the cell cycle.

This review will discuss the current understanding of the role protein arginine methylation plays in cell cycle regulation and the implications for potential cancer treatment.

## Cell division

**Fig. 1:**
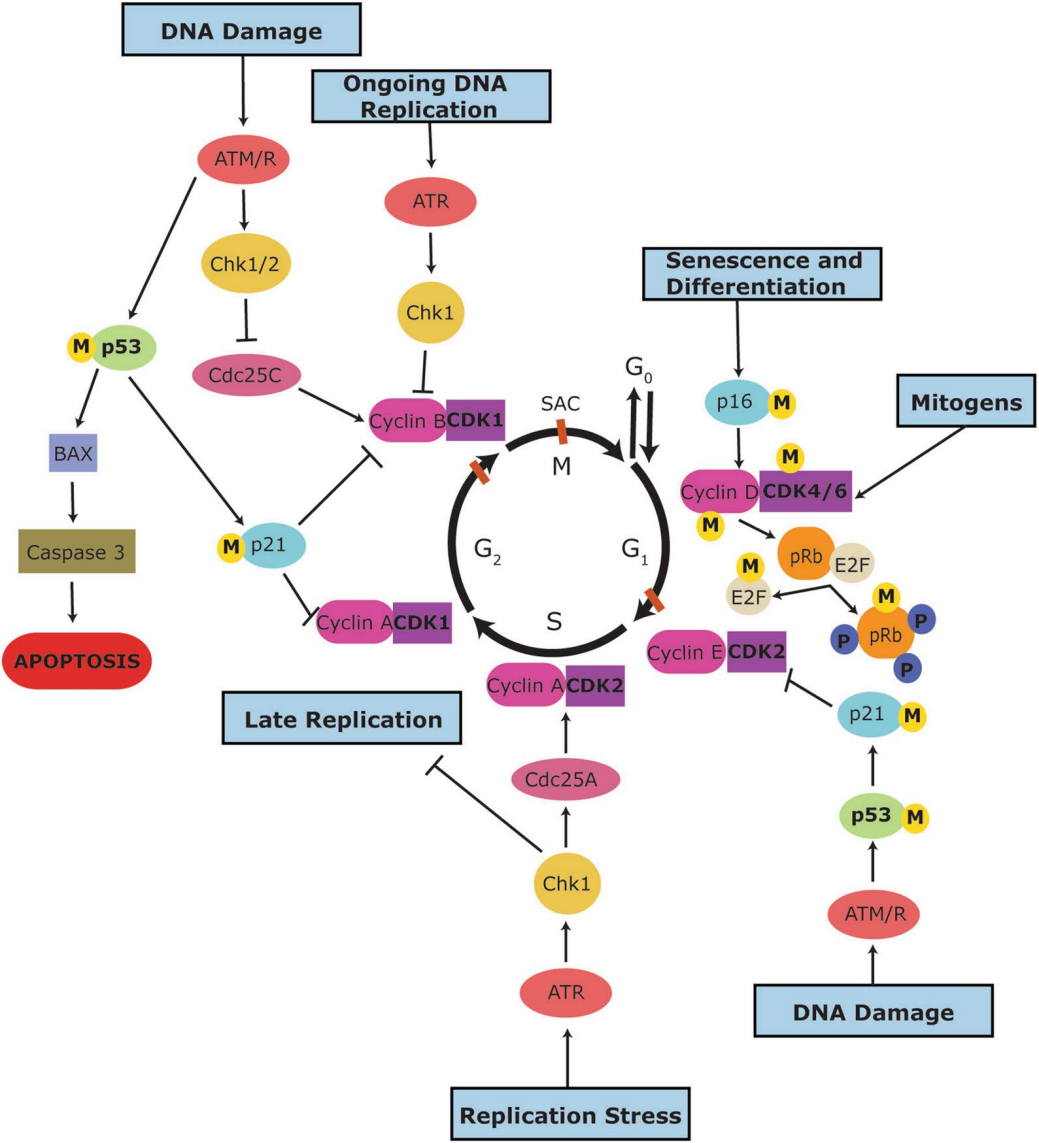
Overview of the regulation of the cell cycle. The cell cycle is depicted as a circle where each black arrow represents one phase of the cell cycle. Cells enter the cell cycle into the G1 phase, which is followed by the S phase, G2 phase and then mitosis (M). The cell cycle is regulated by CDKs (purple), their regulatory subunit cyclins (pink), CDK inhibitors such as p21 (light blue), and other regulatory kinases, such as the checkpoint kinases 1 and 2 (Chk1 and Chk2; yellow). The orange lines at the end of G1, G2 and during mitosis indicate cell cycle checkpoints where the cell is monitored for defects during replication and can respond by the p53 (green) pathway or the ATM/R (red) pathway, among others. Yellow circles with “M” indicate proteins known to be methylated on arginine residues and the dark blue circles with “P” indicate phosphorylation of pRB

The controlled regulation of cell division is essential in growth, repair and re-generation of healthy tissues and occurs via the cell cycle. The cell cycle is divided into non-overlapping stages or phases which are referred to as gap 1 (G_1_), synthesis (S), gap 2 (G_2_) and mitosis (M) phase [5] (Fig. 1). In the G_1_ phase, the cell is preparing for DNA replication which then occurs in the S phase, while cells in the G_2_ phase are preparing for the M phase, where cell division takes place [5]. M phase can be further divided into prophase, metaphase, anaphase, telophase and cytokinesis [6]. For a visual review of mitotic regulation see [7]. Cells that are not actively dividing are said to be in G_0_, a resting or quiescent phase. Once cells have committed to DNA replication they cannot return to G_0_ [1]. In order to guarantee successful cell division without errors, the cell cycle is stringently regulated. Deregulation of the cell cycle, resulting in uncontrolled cell proliferation is one of the hallmarks of cancer [8].

### Cell cycle regulation

Regulation of the cell cycle is mainly achieved by a family of serine/threonine kinases called cyclin-dependent kinases (CDKs). However, full CDK activation only occurs upon the association of a CDK and a cyclin subunit [9]. CDKs allow cell cycle phase transitions to occur [10]. Nine CDKs have been identified with five of them found to be active as complexes with various cyclin subunits during the cell cycle [11, 12]. CDK levels remain constant throughout the cell cycle, while the levels of cyclin proteins rise and fall during the cell cycle to activate CDKs when they are required [13]. Cyclin D1 is overexpressed in 50% of breast cancers [14], and at lower frequencies in many other cancers including prostate cancer [15]; while Cyclin E has been reported to be deregulated in malignant tumors of the lung, breast, gastrointestinal tract and in ovarian cancer [16].

Although CDK levels remain constant throughout the cell cycle, their activity is regulated by phosphorylation on specific residues to induce conformational changes and enhance cyclin binding. For example, full activation of CDK2 requires phosphorylation on threonine 160 by the CDK7-cyclin H complex also called the CDK activating kinase (CAK) [17]. Inhibitory kinases, such as Wee1 and Myt1 can function as inactivators of CDK1 by phosphorylating it. Dephosphorylation is then required for CDK1 reactivation [18]. Cyclin dependent kinase inhibitors (CDKIs) regulate CDK activity by binding to CDKs or the CDK-cyclin complex [1]. There are two families of CDKIs which have been discovered so far: Inhibitors of CDK4 (INK4) proteins and CDK interacting proteins/kinase inhibitory proteins (CIP/KIP) [1] (Fig. 1). The INK4 inhibitors (p15^INK4b^, p16^INK4a^, p18^INK4c^, p19^INK4d^) specifically inactivate the cyclin D-dependent CDKs, i.e. CDK4 and CDK6, by competitively binding these CDKs and preventing them from forming a complex with cyclin D [19]. The CIP/KIP inhibitors (p21^CIP1^, p27^KIP1^, p57^KIP2^) bind to and inactivate multiple G1 CDK-cyclin complexes [20]. p21 also binds to the proliferating cell nuclear antigen (PCNA), inhibiting DNA replication [21]. CDKIs are themselves regulated by internal and external signals. For example, the p21 gene promoter encodes a p53 binding site allowing p53 to transcriptionally activate the p21 gene [22]. The expression of p15 and activation of p27 both increase in response to transforming growth factor β [20]. CDK inhibitors, such as p15 and p18, are frequently deregulated in cancer [3], allowing uncontrolled progression into S phase [23].

The Phosphatidylinositol 3-kinase (PI3K) pathway regulates cellular processes through its association with the protein kinase AKT and is involved in the G_1_/S phase transition of the cell cycle [24]. AKT prevents cyclin D1 degradation by directly phosphorylating glycogen synthase kinase 3 β (GSK3β), blocking its kinase activity and allowing cyclin D1 to accumulate [25]. (PI3K)/AKT signaling is frequently deregulated in human cancers, including ovarian, breast, lung, thyroid and melanomas [26]. The tumor suppressor Phosphatase and tensin homolog (PTEN) gene is a negative regulator of the PI3K/AKT signaling pathway [27] and is one of the most mutated or deleted genes across different cancer types [28], leading to deregulation of the PI3K/AKT pathway.

### Cell cycle checkpoints

In addition to the cell cycle regulation by CDKs and CDKIs, there are several checkpoints, such as the DNA repair checkpoints that regulate G_1_/S and G_2_/M phase transitions (see Fig. 1), where cell size, extracellular growth signals [3], and progression through the cell cycle are monitored to prevent defects or to repair any DNA damage which may have occurred during DNA synthesis [29]. It is these DNA defects, which if left unrepaired, are passed onto daughter cells during cell division and contribute to the deregulation of the cell cycle and may lead to the unrestrained cell proliferation characteristics of cancer [1].

DNA damage checkpoints occur before cells enter S phase (G_1_-S checkpoint) where cell cycle arrest induced by DNA damage is p53 dependent; or after DNA replication, with or without the tumour suppressor protein, p53 (G_2_-M checkpoint) [30]. The checkpoint kinase proteins, Chk1 and Chk2, phosphorylate the cell division cycle phosphatases, Cdc25, to regulate the phosphorylation of CDK1 and CDK2, and therefore regulate G_1_ into S phase and S phase into G_2_/M transitions, respectively. DNA damage repair is also monitored during S phase to block replication, if damaged DNA has escaped the G_1_/S phase checkpoint without undergoing repair [31].

The spindle assembly checkpoint (SAC) detects the improper alignment of chromosomes on the mitotic spindle and can stop the cell cycle in metaphase, if required, to prevent the formation of cells with the incorrect number of chromosomes [32]. This checkpoint is controlled by the mitotic-arrest deficient (*mad*) and the budding uninhibited by benzimidazole (*bub1*) families of genes [33, 34]. The mitotic checkpoint complex (MCC) consists of MAD2, BUBR1 and BUB3 proteins and negatively regulates the activity of cell division cycle protein 20 (CDC20) [35–38]. CDC20 binds to the ubiquitin ligase anaphase-promoting complex/cyclosome (APC/C) [38], preventing the APC/C from exerting its ubiquitinylation activity on securin and cyclin B [39]. Securin is an inhibitor of the protease separase which is required to cleave the cohesion complex which holds sister chromatids together [40], while cyclin B is required to activate CDK1 to promote exit of the cells from mitosis [41]. Thus, inhibition of the APC/C by the MCC prevents cells from entering anaphase. Other important SAC proteins include MAD1, BUB1, MAPK and Aurora B, which promote recruitment of the SAC proteins to the kinetochores [42]. Aurora B is also involved in correcting aberrant merotelic attachment of kinetochores to microtubules from opposite poles to prevent the division of cells with the improper number of chromosomes [43]. Ubiquitin-associated protein 2-like (UBAP2L) is also necessary for proper kinetochore/microtubule attachment and depletion of UBAP2L activates SAC signalling, delaying progression into anaphase [44]. For a comprehensive review on the SAC see [45].

### DNA damage repair

DNA damage can be induced via a variety of triggers (including UV radiation, chemicals, and stress) and if left unrepaired can lead to cancer. Therefore, cells respond to unrepairable DNA damage by stopping cell cycle progression or by initiating programmed cell death via several pathways.

Depending on the type of DNA damage, five major pathways of DNA repair are utilised by cells including: direct repair, base excision repair, nucleotide excision repair, mismatch and recombinational repair, and double strand break repair [46]. These repair pathways will be briefly explained here but the reader is referred to other reviews [47–51] for a more comprehensive coverage.

In mammalian cells, alkylation damage is repaired by direct repair alkyltransferases. O^6^-methylguanine-DNA methyltransferase is the main enzyme facilitating direct repair by transferring a methyl group from the DNA backbone into the active site of the enzyme [52].

Three excision repair mechanisms remove specific types of damage: Firstly, base excision repair (BER) specifically recognizes base damage. It most commonly deals with base damage that occurs during normal aerobic metabolism via reactive oxygen species (most common base damage: dihydro-8-oxoguanine). BER requires removal of the damaged DNA base by a specific DNA glycosylase to initiate the process [51]. Short nucleotide gaps are then re-synthesized via APE1, DNA polymerase β [53] and the Ligase 3/XRCC1 complex; while longer breaks of 2–10 nucleotides are repaired by the RFC/PCNA-DNA polymerase δ/ε complex [54]. Secondly, nucleotide excision repair is the major pathway for the removal of bulky DNA lesions formed by exposure to environmental sources, ultraviolet radiation or chemicals [55]. In humans, the process of nucleotide excision repair involves damage recognition, incision around the lesion to release a 24–32 nucleotide oligomer and repair of the resulting gap by DNA polymerase δ/ε and ligation. For a review on nucleotide excision repair see [56]. In humans, nucleotide excision repair requires more than 30 proteins to successfully execute the repair [55]. Repair proteins including XPA, RPA, XPC, TFIIH, XPG and XPF-ERCC1 perform these functions [57–59]. Thirdly, mismatch repair functions to repair errors such as mismatches, insertions or deletions that can occur during replication [50].

Double strand breaks (DSBs) are the most lethal type of damage to the cell. DSB damage occurs when cells are exposed to ionizing radiation and during normal cellular recombination and immunoglobulin class-switching processes [60]. DSBs can be repaired via homologous recombination or non-homologous end-joining (NHEJ) [61]. Homologous recombination occurs at a homologous stretch of DNA on a sister chromatid serving as a template to guide repair of the broken strand, while NHEJ requires enzymes to capture the ends of the broken DNA, bringing them together in a DNA–protein complex, then the DNA break is repaired via ligation [62]. Both require the proteins NBS1 [63], MRE11 [64], and Rad50 [65]. Homologous recombination also requires the heterodimer MUS81–MMS4 [46], BRCA1 and BRCA2 [66], while NHEJ requires DNA-PKCs and the heterodimer Ligase 4-XRCC4 [67].

DNA strand crosslinks can be induced by many chemotherapeutic drugs and can stall DNA replication, leading to cell death [68]. Depending on the type of cross-link, they are processed either by nucleotide excision repair (intra-strand cross-link) or converted into a double-stranded DNA break and processed by homologous recombination (inter-strand crosslink) [69]. Hence, the same repair proteins are utilised, such as XPF-ERCC1 to degrade one of the cross-linked strands [70]; or the recombination proteins Rad51 and Rad52 to promote homologous DNA pairing and strand exchange [65].

DNA damage repair can also be initiated by tumor suppressor proteins which can induce cell cycle arrest to either initiate repair or mark the cell for apoptosis. Two such proteins that regulate the major pathways of apoptosis and cellular progression are p53 and pRb [10]. Between them, these two proteins can initiate differentiation, DNA repair, cell cycle arrest or progression and induction/inhibition of apoptosis. The p53 and pRb pathways are summarised in the following sections.

### p53

p53 is normally expressed at low levels but rises within cells in response to various stimuli, such as DNA damage, hypoxia and oncogene activation, to activate various pathways that initiate differentiation, DNA repair, cell cycle arrest, inhibition of angiogenesis and apoptosis [71]. There are three main responses that p53 can initiate: (1) facilitation of cell cycle arrest by inducing the expression of p21, 14-3-3 proteins, Cdc25C and GADD45 [72]; (2) stimulation of DNA repair by inducing the expression of p21, GADD45 and p48 [10]; and (3) induction of apoptosis by upregulating the transcription of BAX and other apoptotic proteins [73]. Low levels of stress or DNA damage induce p53-mediated cell cycle arrest, while high levels of stress activate p53-mediated apoptotic pathways [74]. Further, different protein kinases such as ataxia-telangiectasia mutated (ATM) and ataxia-telangiectasia and Rad3 related (ATR) phosphorylate p53 in response to DNA damage, leading to p21 arresting the cell cycle at the G_1_-S checkpoint [47, 75] (see Fig. 1). Inactivation of the p53 gene results in the dysfunction of proteins that would normally inhibit cell cycle proliferation [1]. The inactivation of the p53 gene through mutations is the most commonly occurring loss of a pro-apoptotic regulator and is seen in more than 50% of cancers [76].

### Retinoblastoma protein

pRb interacts with proteins involved in transcriptional control such as the E2F proteins that are regulators of gene expression and genes involved in DNA replication, DNA repair and G_2_/M progression [77] (Fig. 1). pRb regulates cell cycle arrest by binding to E2F. E2F and its heterodimer, the transcription factor DP, are mediators of the p16/pRb pathway of G_1_ cell cycle arrest [78]. However, only unphosphorylated forms of pRb can interact with E2F proteins [79] (Fig. 1). Thus, phosphorylation of pRb carried out by the CDK4-cyclin D1 complex regulates pRb function [80]. pRb is found to be unphosphorylated during G_0_ and G_1_ phase, and phosphorylated during the remainder of the cell cycle [81].

Phosphorylation clearly plays a pivotal role in cell cycle regulation. Protein methylation is a post-translational modification that may be just as important in cell cycle regulation as phosphorylation.

## Protein arginine methylation

During protein methylation, a methyl group is added to a specific protein residue to alter detection by its binding partners or to regulate its activity [82]. This can occur on lysine (K), histidine (H) or arginine (R) residues of both histones and non-histone proteins [83]. Protein arginine methyltransferases were discovered almost 50 years ago and while histone and non-histone protein lysine methylation have been extensively studied over the past 60 years, protein arginine methylation has only gained more attention in the past 20–25 years [84].

Protein arginine methylation has many documented regulatory roles including in signal transduction [85], transcription [86, 87], protein–protein interactions such as facilitating the interactions of Tudor domains with glycine arginine rich (GAR) and proline glycine methionine (PGM) motifs in proteins [88], RNA transport [89], and RNA splicing [90, 91]. For a recent review that covers arginine methylation in different organ systems, see [92].

### Protein arginine methyltransferases

**Fig. 2:**
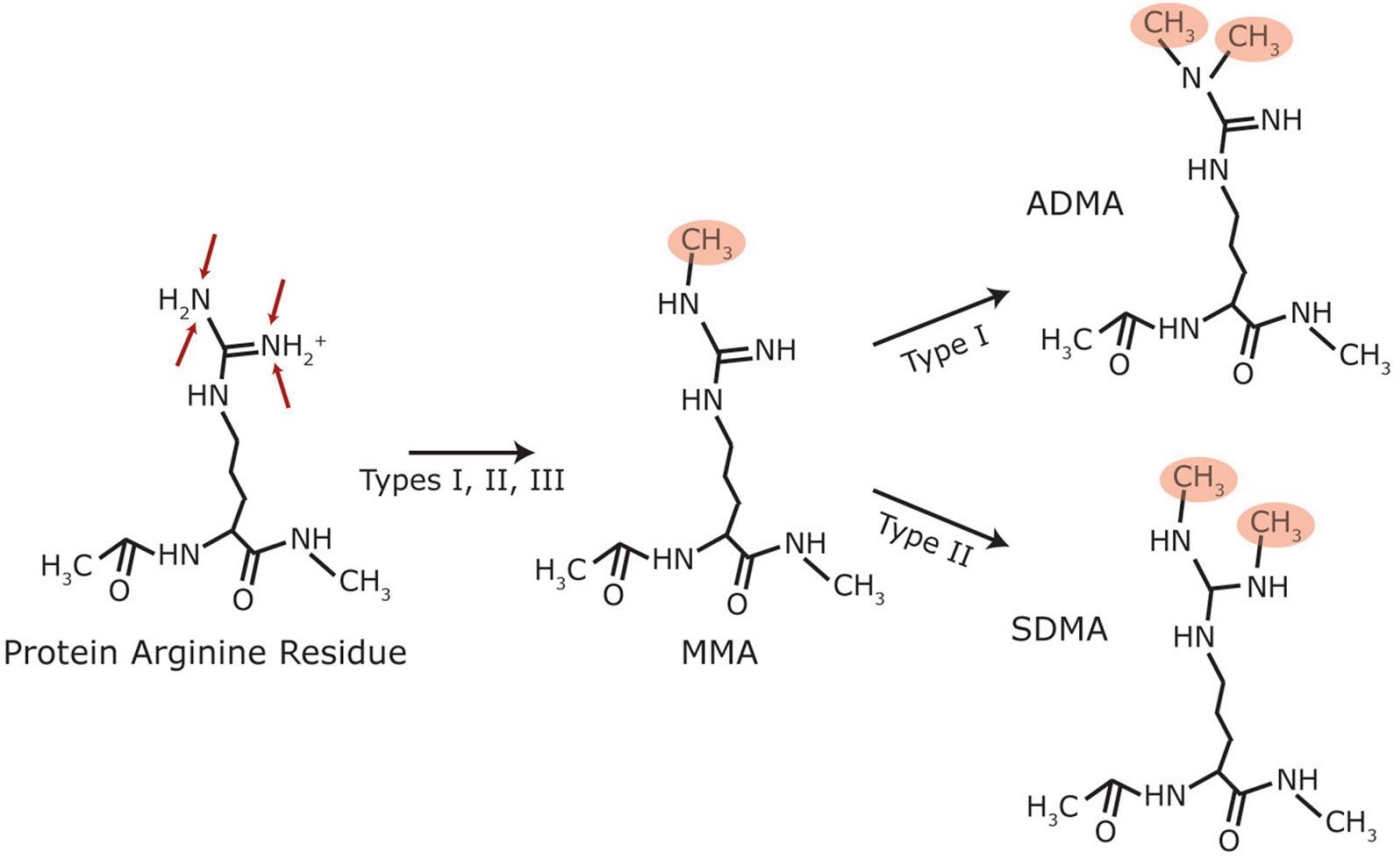
Types of mammalian protein arginine methylation. Arginine residues in proteins can be monomethylated by type I, II and III PRMTs to form MMA, while type I and II PRMTs further methylate to produce ADMA and SDMA residues, respectively. Red arrows indicate known methylation locations in mammalian cells; red circles indicate methyl groups

In 1968, Paik and Kim attempted to purify the enzyme responsible for the methylation of histone lysine residues [93]. The enzyme they discovered, protein methylase I, was not a lysine methyltransferase but actually a protein arginine methyltransferase or PRMT [93], now known as PRMT1. There are currently eleven PRMTs which are divided into four enzyme types [94]. For a review see [82, 95, 96]. Types I to III exist in mammalian cells and all catalyse the addition of a single methyl group onto a terminal nitrogen atom of arginine residues forming ω-*N*^G^-monomethyl arginine (MMA). Type I enzymes are the most common [4] and catalyse the addition of another methyl group onto the same terminal nitrogen atom, forming ω-*N*^G^, *N*^G^-dimethylarginine or asymmetric dimethylarginine (ADMA) [97]. Type II enzymes catalyse the addition of another methyl group onto the other terminal nitrogen atom, producing ω-*N*^G^, *N*’^G^-dimethylarginine or symmetric dimethylarginine (SDMA) [98]. Type III enzymes only catalyse the formation of MMA [99]. While Type IV enzymes also catalyse the formation of MMA, the methyl group is added to the internal (σ) guanidino nitrogen atom and this has so far only been described in yeast [100]. The structures of the three methylarginine types present in mammals (MMA, ADMA, and SDMA) are shown in Fig. 2.

### Regulation of the cell cycle by arginine methylation

The interaction of many key regulatory proteins of the cell cycle with several PRMTs has been characterised and/or methylated residues have been identified (for summary see Table 1). It should be noted that these findings, while representative of the cell type and/or tissue types examined, may not be representative of all tissues especially as cancer is notorious for differing mutations between patients. While there had been earlier indications that arginine methylation may be involved in cell proliferation, a study by Kim et al. [101] was one of the first to describe the involvement of protein arginine methylation in the different phases of the cell cycle in HeLa cells.

**Table 1:** Key cell cycle regulators known to be substrates and interacting partners of PRMTs

Protein	Interacting PRMT	Known methylated residues	Result of methylation or PRMT interaction	Refs.
BGT2	PRMT1	–	BGT2 regulates PRMT1 activity in pre-B cells	[103]
CDK4	PRMT1	R55, R73, R82, R163	Destabilization of CDK4-cyclin D3 complex leading to pre-B cell differentiation	[103]
	PRMT5	–	Interaction of CDK4 and PRMT5 regulates pRb/E2F-mediated transcription	[119]
Cyclin D1	PRMT2	–	Knockdown of PRMT2 correlates with increased cyclin D1	[111]
	PRMT5	–	Increased nuclear PRMT5 correlates with increased cyclin D1 protein levels	[121]
E2F1	PRMT1	R109	Assists E2F1-dependent apoptosis during DNA damage	[104, 105]
	PRMT4	–	Regulates E2F expression	[113]
	PRMT5	R111, R113	Promotes cell proliferation	[104, 106]
Fanca	PRMT5	–	Regulation of PRMT5-mediated methylation of p53	[131]
p16	PRMT1	R138	Regulates p16 and CDK4 interaction to regulate cell proliferation	[107]
	PRMT5	–	Increased nuclear PRMT5 negatively correlates with p16 protein expression and is associated with poor survival	[121]
	PRMT6	R22, R131, R138	Methylation of p16 reduces binding of p16 with CDK4	[136]
p21	PRMT2	–	Knockdown of PRMT2 correlates with increased p21 expression	[110]
	PRMT6	–	Inhibition allowing cell cycle progression	[132]
		R156	Increased cytoplasmic localisation of p21, resulting in resistance to doxorubicin	[135]
p27	PRMT6	–	Inhibition allowing cell cycle progression	[132]
p53	PRMT5	R333, R335, R337	Affects promoter specificity of p53 and enhances p53-dependent cell cycle arrest	[127]
	Unknown	R213	Mediates p21 activation for cell cycle progression	[129]
pRb	PRMT2	–	Repression of E2F transcriptional activity and cell cycle progression by binding to pRb	[108]
	PRMT4	R775, R787, R798	Decreases binding of pRb to E2F-1 leading to increased cell proliferation	[109]

#### PRMT1

As the most abundant PRMT [102], the majority of PRMT1 activity can be described as anti-proliferative and/or tumor-suppressor-inducing. Recent work has revealed that PRMT1 binds to the tumor suppressor, BGT2, to play a role in pre-B cell differentiation [103]. The BGT2-PRMT1 complex destabilizes the binding of CDK4 to cyclin D3 through the methylation of CDK4 at arginines 55, 73, 82 and 163, halting cell cycle progression and leading to pre-B cell differentiation [103]. E2F1 is competitively methylated by PRMT1 and PRMT5 [104]. Methylation by PRMT1 stabilizes E2F1 during DNA damage [105] and assists in E2F1-dependent apoptosis [104], while methylation by PRMT5 promotes cellular proliferation [104, 106]. Interestingly, recent publications have brought to light the interplay between arginine methylation and serine phosphorylation, adding an extra layer of complexity to the regulation of the cell cycle. For example, methylation of p16 at arginine 138 by PRMT1 increases as phosphorylation of p16 at serine 140 decreases, and vice versa in 293T cells [107]. These modifications work antagonistically to regulate the interaction of p16 with CDK4 [107], thus regulating cell proliferation and apoptosis.

#### PRMT2

It is unclear at this stage whether PRMT2 has pro-proliferative or anti-proliferative functions in cancer. Arginine methylation of pRb by PRMT2 contributes to E2F1 transcriptional regulation for progression into S phase [108] by impairing pRb/E2F1 binding [109]. Recently, PRMT2 levels were found to be decreased in breast cancer cells, while knockdown of PRMT2 correlated with increased expression of both p21 [110] and cyclin D1 [111] indicating a regulatory role for PRMT2 in cell cycle progression. Further studies are needed to clarify the exact functions of PRMT2 in cell cycle progression.

#### PRMT4

PRMT4 appears to have pro-proliferative functions and may have a pro-oncogenic role in some cancers. Expression of Cyclin E1 rises sharply leading into S phase to allow transition into the S phase [6], and levels of cyclin E1 are frequently deregulated in breast cancer [16]. PRMT4 is a positive regulator of the Cyclin E1 gene, by acting as a transcriptional co-activator of ACTR coinciding with histone methylation of the CCNE1 promoter region [112]. PRMT4 is essential for estrogen induced cell cycle proliferation in breast cancer by the positive regulation of E2F1 RNA and protein expression [113]. In addition, increased expression of PRMT4 may contribute to the development of prostate cancer, as it correlates with the androgen independence required for the progression of aggressive tumours [114]. Interestingly, a decrease in PRMT4-mediated methylation of pRb led to a decrease in its phosphorylation, suggesting arginine methylation can increase pRb-mediated cell proliferation [109].

#### PRMT5

The most studied type II PRMT, PRMT5, has pro-proliferative and pro-oncogenic roles, as described below, even though it appears to have the opposite effect in Fanconi anemia patients. This highlights that the effect of specific PRMTs and their inhibition may not be the same in all types of cancers and may differ in individual patients.

PRMT5 is essential for cell proliferation [115] and correlates with increased protein expression of the G_1_ phase regulators CDK4 and CDK6 [116]. Deficiency of PRMT5 triggers cell-cycle arrest in the G_1_ phase [117]. Overexpression of PRMT5 correlates with increased cell proliferation and knockdown of PRMT5 results in cell cycle arrest leading to apoptosis [118]. CDK4 interacts with PRMT5 in HepG2 cells which regulates phosphorylation of pRb, thus regulating pRb/E2F-mediated transcription [119]. Disruption of the PRMT5/CDK4 interaction revealed PRMT5 knockdown HepG2 cells to be more sensitive to the CDK4 inhibitor fascaplysin, marking the combination of PRMT5 and CDK4 inhibition as a potential cancer therapy [119]. PRMT5 deficiency also led to apoptosis in differentiated glioblastoma cells however, in glioblastoma neurospheres it led to G_1_ cell cycle arrest through increased protein expression of p27 and a decrease in phosphorylation of pRb [120]. PRMT5 expression correlated with cyclin D1 protein levels, while also inversely correlating with p16 levels [121]. Nuclear PRMT5/p16-negative tumors were associated with poor prognosis in oropharyngeal squamous cell carcinoma when compared to nuclear PRMT5-negative/p16- positive tumors [121]. PRMT5 is found to be overexpressed in many cancer types, including glioblastoma [118, 122], lung [123], mantle cell lymphoma [124], ovarian [125] and prostate cancer [126]. In all these studies, knockdown of PRMT5 slowed or inhibited cell proliferation, indicating the enzyme may be affecting key regulators of cell proliferation.

p53-dependent cell cycle arrest is enhanced by methylation of p53 by PRMT5 [127], while reduced expression of p53 during the DNA damage response is triggered by PRMT5-deficiency [117]. While the methylation of p53 at arginine 213 may be controversial [128], mutation of this residue resulted in a decrease of p21 RNA and protein expression directly affecting the S phase of the cell cycle [129]. This indicates that p21 activation by p53 may be mediated by arginine methylation of R213 [129].

Fanconi anemia is a genetic disorder characterized by a high risk of developing cancer, among other clinical characteristics, due to a cellular hypersensitivity to DNA cross-linking agents and faulty DNA damage repair pathways [130]. Fanconi anemia proteins, FANCA-C, E–G, L and M, form a “core complex” which activates DNA repair pathways. In *Fanca*^−*/*−^ and *Fancc*^−/−^ mice, arginine methylation of p53 by PRMT5 was decreased in response to oncogenic stress [131]. Interestingly, forced expression of PRMT5 led to the delayed onset of leukemia in irradiated mice [131], indicating that PRMT5 may play a tumor suppressor role in Fanconi anemia patients.

#### PRMT6

PRMT6 may have pro-proliferative and pro-oncogenic functions in both colon and lung cancers. PRMT6 inhibits p21 along with p27, allowing cell cycle progression through the cyclin dependent kinases 1 and 2 (CDK1/2) [132–134]. Methylation at arginine 156 of the p21 protein by PRMT6 increased the cytoplasmic localisation of p21 and resulted in HCT116 colon cancer cells becoming more resistant to the chemotherapy drug, doxorubicin [135]. Methylation of p16 at arginine 138 by PRMT6 caused reduced binding of p16 to CDK4, leading to increased cellular proliferation in A549 cells [136]. Further studies are required to determine the function of PRMT6 in other types of cancers.

### Regulation of the DNA damage repair by arginine methylation

Proteins involved in the DNA damage repair pathways are regulated by protein arginine methylation (see Table 2). Arginine methylation of Heterogeneous Nuclear Ribonucleoprotein U-Like 1 (hnRNPUL1) by PRMT1 regulates the interaction of hnRNPUL1 with Nijmegen Breakage Syndrome 1 (NBS1) [137], a component of the double strand DNA break repair complex with MRN (MRE11/Rad50/NBS1). Arginine methylation of hnRNPUL1 also regulates its recruitment to sites of DNA damage [137]. The DNA damage repair proteins, MRE11 and p53 binding protein (p53BP1), are methylated by PRMT1 which regulates their DNA exonuclease activity [138, 139] and localisation to DNA damage sites [140], respectively. PRMT1-deficient cells have an impaired ability to recruit Rad51 to DNA damage sites, causing chromosome instability and cell cycle arrest [141]. PRMT1 also regulates the activity of DNA polymerase β by methylating arginine 137 within the PCNA binding site [142]. This prevents the binding of DNA polymerase β and PCNA, implicating PRMT1 in regulation of BER specifically.

**Table 2:** Key DNA damage repair proteins known to be substrates and interacting partners of PRMTs

Protein	Interacting PRMT	Known methylated residues	Result of methylation or PRMT interaction	Refs.
DNA polymerase β	PRMT1	R137	Regulates binding to proliferating cell nuclear antigen	[142]
	PRMT6	R83, R152	Enhances DNA binding affinity of DNA polymerase β and enhances repair ability	[144]
FEN1	Unknown	R192	Enhances localization to DNA repair sites and binding to PCNA	[145]
hnRNPUL1	PRMT1	R584, 5618, R620, R645, R656	Regulates interaction with NBS1 and recruitment to DNA damage site	[137]
MRE11	PRMT1	–	Regulation of DNA exonuclease activity	[138, 139]
p53BP1	PRMT1	R1400, R1401, R1403	Enhanced localisation to DNA damage sites	[140]
Rad9	PRMT5	R172, R174, R175	Regulation of checkpoint activation	[143]

PRMT5 also regulates DNA damage repair through the methylation of Rad9 [143]. HEK293T cells without methylated Rad9 were more susceptible to DNA damage by hydroxyurea and led to increased S/M and G_2_/M cell cycle checkpoint activation [143].

The DNA binding affinity of DNA polymerase β and its ability to repair short single-stranded DNA breaks is enhanced by the methylation of DNA polymerase β by PRMT6 [144].

Methylation of DNA repair protein, Flap endonuclease (FEN1) at arginine 192, enhances its localization to DNA repair sites [145]. Interestingly, a disruption in arginine methylation causes reduced binding to PCNA allowing the phosphorylation of FEN1 at serine 187 by the CDK2/cyclin E complex, leading to decreased localization of FEN1 at DNA repair sites and a delay in cell cycle progression [145].

### Regulation of indirect mediators of the cell cycle by arginine methylation

In addition to the key regulatory proteins of cell cycle and DNA damage repair pathways discussed in the previous sections, other cellular proteins that can indirectly affect cell cycle progression are regulated by protein arginine methylation (see Table 3).

**Table 3:** Indirect mediators of the cell cycle known to be substrates and interacting partners of PRMTs

Protein	Interacting PRMT	Known methylated residues	Result of methylation or PRMT interaction	Refs.
Androgen receptor	PRMT2	–	Co-activator allowing translocation into the nucleus	[152]
	PRMT5	–	Activator of the AR	[154]
	PRMT10	–	Knockdown of PRMT10 suppressed cell growth in LNCaP cells	[153]
CREB-binding protein	PRMT4	R600	Disrupts CREB binding	[155]
		R742	Regulates transcriptional activation of steroid hormone receptors	[156]
Estrogen receptor α	PRMT1	R260	Cytoplasmic localisation of ERα prevents phosphorylation of PKB/AKT	[148]
	PRMT2	–	Co-activator of ERα, implicated in tumour cell growth	[149]
INCENP	PRMT1	R887	Enhances binding with inner centromere protein (INCENP) to regulate chromosomal alignment and segregation	[147]
MDM4	PRMT5	–	Alternate splicing of MDM4 activates p53 in response to PRMT5 depletion	[172]
p300	PRMT4	R580	Methylation of p300 activates p21 to inhibit cell cycle progression	[155]
		R754	PRMT4 complexes with p300, BRCA1 and p53 to induce expression of p21	[157]
Sam68	PRMT1	R45, R52, R304, R310, R315, R320, R325	Methylation of Sam68 regulates its localization and reduces its RNA-binding ability	[169] [170]
	PRMT2	–	Regulates alternative splicing of Bcl-x	[168]
SF2/ASF	Unknown	R93, R97, R109	Regulates subcellular localization and activity of SF2/ASF	[171]
Telomere repeat binding factor 2	PRMT1	R17, R18	Regulates telomere length and stability	[146]
Ubiquitin-associated protein 2-like	PRMT1	N-terminus region	Regulation of chromosome alignment during mitosis	[44]

PRMT1 regulates telomere length and stability by the methylation of the Telomere repeat binding factor 2 (TRF2) [146]. Depletion of PRMT1 results in telomere doublets and promotes telomere shortening [146]. Methylation at arginine 887 of the Inner centromere protein (INCENP) by PRMT1 enhances its binding affinity to Aurora B [147]. A decrease in INCENP methylation led to repression of Aurora B activity resulting in abnormal chromosome alignment and segregation [147]. Arginine methylation of Ubiquitin-associated protein 2-like (UBAP2L) by PRMT1 is necessary for progression of mitosis by regulating chromosome alignment and distribution [44].

The estrogen receptor α (ERα) is methylated by PRMT1 at arginine 260, resulting in cytoplasmic localisation of ERα and indirectly prevents the downstream phosphorylation of the protein kinase β/α serine/threonine-protein kinase, PKB/AKT [148]. PRMT2 is another co-activator for the ERα and has been implicated in tumour cell growth and progression [149].

The androgen receptor (AR) found in the prostate plays a role in regulating the G_1_/S phase transition of the cell cycle in prostate cancer [150]. Inhibition of arginine methylation resulted in reduced expression of the AR and reduced cell proliferation [151]. PRMT2 acts as a co-activator of the AR, allowing translocation of both the AR and PRMT2 from the cytoplasm into the nucleus [152]. The AR also associates with PRMT9/10 in the prostate cancer cell line, LNCaP, and knockdown of PRMT9/10 suppressed both cellular growth and expression of the prostate specific antigen (PSA) [153]. PRMT5 was recently found to function as an activator of the AR and regulator of AR-dependent proliferation in LNCaP cells and expression of PRMT5 also correlated with RNA and protein expression of the AR [154]. Although it is not yet known if the AR is methylated on arginine residues, it is clear that methylation plays an important role in AR-mediated cell cycle progression, through the association with PRMTs.

The transcriptional activity of the acetyltransferases CREB-binding protein (CBP)/p300 is regulated by PRMT4-mediated methylation of p300, which is important for G_1_/S phase transition [155, 156]. Methylation of p300 induces complex formation of PRMT4 and p300 with BRCA1 and p53 to induce expression of p21, hence inhibiting cell cycle progression [155, 157]. p300 also associates with PRMT5, Strap and JMY for arginine methylation of p53 which affects the promoter specificity of p53 and enhances p53-dependent cell cycle arrest [127].

Pathways involved in cell cycle regulation can also affect alternative splicing. The ATM/ATR pathways can regulate alternative splicing in response to DNA damage to promote pro-apoptotic genes [158]. Aurora kinase A inhibition caused downregulation of the splicing factor, SF2/ASF, to regulate bcl-x splicing to trigger apoptosis [159]. Interestingly, splicing factors and their products are often overexpressed in cancer, including ovarian [160], breast [161, 162], lung and colon [161]. hnRNPs are a prominent splicing factor family known to be methylated on arginine residues [163–165]. Serine–arginine rich (SR) splicing factors are another prominent splicing factor family [166] and it should come as no surprise that SR splicing factors are methylated due to the abundance of arginine residues they contain. The SR protein SF2A-p32 associates with PRMT1 and PRMT5 [167], while PRMT2 associates with multiple SR proteins and hnRNPs, including Sam68, to regulate alternative splicing of the mitochondrial protein Bcl-x [168]. Arginine methylation of Sam68 by PRMT1 localizes it to the cytoplasm [169] and reduces its RNA binding ability [170], thus indicating that Sam68 is highly regulated by arginine methylation. The localisation and therefore the function of SF2/ASF is also highly regulated by arginine methylation [171]. PRMT4 methylates the splicing factors CA150, SmB, U1C, and SAP49 and promotes exon skipping to regulate alternative pre-mRNA splicing [86]. Alternative splicing of the p53 regulator, MDM4, activates the p53 pathway in response to PRMT5 depletion [172]. The role of PRMTs in splicing has been reviewed by [173]. The effect of the methylation of splicing factors in cell cycle regulation and cancer needs to be further investigated.

From this review, it should have become evident, that the majority of proteins and pathways of the cell cycle reported to be deregulated in various cancers are also the ones that can be regulated via protein arginine methylation. Further, studies that suggest regulation of the DNA damage response by arginine methylation are accumulating [44, 137, 138, 140, 142, 144]. These studies also implicate arginine methylation in the deregulation of DNA damage repair pathways which, if not rectified by the cell, may lead to genomic instability and eventually to the progression of cancer. Altered expression levels of PRMTs have been found in many types of cancer, reviewed by [94, 174], implicating their importance in cell cycle and proliferation, cell cycle checkpoints and DNA replication [175]. Therefore, protein arginine methylation may represent a novel target in the development of anti-cancer drugs.

## Implications for cancer treatment

PRMT1 [176, 177], PRMT2 [178], PRMT4 [179], PRMT5 [180], PRMT7 [181], and PRMT8 [182] have been reported to have isoforms with differing subcellular locations and are expressed in different cancer types. For a review see [183]. Although Baldwin et al. [183] speculate that the cancer-specific isoforms may have different substrates, this is based on the different localisations of the isoforms which would allow access to different substrates [183]. It is currently unknown if these isoforms behave differently in their substrate-specificity or their activity. These cancer-specific PRMT isoforms could also be targets for future anti-cancer drug development. Drugs could potentially target the aberrant isoforms in cancerous cells without affecting the other isoforms required for normal cell function.

The opportunity may further exist to use PRMT inhibitors in combination with classic chemotherapy drugs. The currently widely used chemotherapy drug Taxol (Paclitaxel) binds to tubulin-β, causing stabilisation of microtubules during mitosis and inhibiting cell division [184]. Recently, protein arginine methylation in the Taxol-binding region of tubulin-β was proposed to affect the binding ability of the drug [185]. This could pave the way for the use of methylation inhibitors as a parallel treatment with Taxol. Inhibiting arginine methylation of tubulin-β would allow Taxol full access to the tubulin-β binding site to inhibit cell division in a cancer- targeted therapy. Alternatively, Taxol derivatives that bind with higher affinity to methylated tubulin may be developed.

PRMT inhibitors could also be used in combination with chemotherapy drugs to combat chemoresistance. There are multiple known pathways of chemoresistance (for review see [186]). Activation of the NF-κB pro-inflammatory pathway can activate the production of anti-apoptotic proteins resulting in tumour growth [187]; polymorphisms in ATP-binding cassette (ABC) multidrug efflux pumps can prevent drugs from crossing the blood–brain barrier and even pump drugs back out of target cells [188]; or DNA repair pathways can be upregulated to repair damage caused by DNA-damaging drugs [189, 190]. As discussed previously, PRMT1 methylates or associates with DNA repair proteins and in many cases, this regulates or enhances the binding of DNA repair proteins to the damage sites [137, 138, 140, 142–145]. An inhibitor targeting PRMT1 could be used to disrupt the binding of DNA repair proteins to the DNA damage sites, thus preventing cancer cells from evading apoptosis. This treatment would need to be delivered in a tumor-targeting vector as PRMT1 knockdown is known to be embryonically lethal [141] and would also be damaging to non-tumorigenic cells.

Other drugs should be looked at with the possibility of combinatorial cancer therapies. As discussed previously, PRMT5 interacts with CDK4 to regulate pRb/E2F-mediated transcription. Knockdown of PRMT5 made HepG2 cells more sensitive to the CDK4 inhibitor fascaplysin [119]. While this is a promising study, fascaplysin has only been used on cell lines [191–194], and would require further study in order to be used for cancer therapy. PD0332991 (Palbociclib), was the first CDK4/6 inhibitor to be approved for cancer therapy. It is used in the treatment of pRb positive breast cancer [195], mantle cell lymphoma (MCL) [196], and liposarcoma [197]. This CDK4 inhibitor may also be a potential candidate for combination therapy with PRMTs. An earlier study showed that knockdown of PRMT7 sensitised HeLa cells to the DNA topoisomerase I inhibitor Camptothecin, although the authors did not investigate the mechanism of how this occurs [198]. PRMT7 does not have many known substrates and its PRMT type status has been a controversial topic in the past with some studies claiming it to be a type II PRMT [199], while others proposed it to be a type III PRMT [99]. Further research on the exact mechanism of methylation by PRMT7 is required before it can be considered as a target for combinatorial therapy.

As mentioned previously, PRMTs are often overexpressed in cancers, leading to aberrant methylation patterns. Inhibitors of arginine methylation may be useful to treat tumors by correcting increased levels of protein methylation which may deregulate the cell cycle, DNA damage repair and other important cellular functions. Arginine methyltransferase inhibitor-1 (AMI-1), is able to inhibit the coactivator function of PRMTs [200] while Adenosine dialdehyde (AdOx) inhibits *S*-adenosyl homocysteine hydrolase preventing methylation from occurring by a negative feedback mechanism [89]. Although AdOx does not inhibit PRMTs directly, several studies have shown that treatment of cell lines with high concentrations of AdOx induces a G_2_/M phase arrest of the cell cycle [201–203]. Inhibition of methylation with AdOx also showed a similar decreased growth rate and reduced migration activity when compared to PRMT1 knockdown cells [204]. This may be useful in treating tumors, if the inhibition of methylation by AdOx can be targeted towards tumor cells only. However, AdOx also causes a decrease in DNA, RNA and lysine methylation, thus further studies would be required to determine its suitability for treatment in vivo as well as the actual role of DNA, RNA and lysine methylation in cancer development.

Other non-specific PRMT inhibitors have been identified-MS023 binds to the active site of type I PRMTs [205]; while DS-437 acts on PRMTs 5 and 7 [206]. Inhibitors which are more specific to individual PRMTs would be preferential for combinatorial cancer therapy to minimise potential off-target effects. Promising specific PRMT inhibitors will be discussed below and have recently been reviewed [207].

PRMT1, being the most abundant PRMT [102], has been the target of the majority of PRMT-specific inhibitor development. Cyanine-derivative compounds have been synthesized based on the structure of AMI-1, such as E-84 which preferentially binds to PRMT1 over other PRMTs and has been demonstrated to decrease leukemia cell proliferation [208]. High-throughput screening was utilised to identify compounds to competitively bind to PRMT1 and PRMT8 [209]. Only PRMT1 and PRMT8 contain a hyper-reactive cysteine residue (C101) within the active site that comes into direct contact with S-adenosyl methionine during the methylation reaction [210]. CID5380390 and CID2818500 were found to produce the strongest inhibition with IC_50_ values of 23 and 11 μM, respectively [209]. CID5380390 was used to characterize PRMT activity in *E. grandis* roots [211]. However, no further studies have been published on the mechanism of action for either of these inhibitors.

The PRMT5-specific inhibitor, EPZ015666 (GSK3235025), inhibited growth in a panel of five MCL cell lines and inhibited tumor growth in a dose-dependent manner in MCL xenograft models [212]. The inhibitor structure was altered and renamed EPZ015938 and entered phase I clinical dose-escalation trials in 2016 as compound GSK3326595 for the treatment of solid tumors and non-Hodgkin’s lymphoma (NCT02783300) [213].

More specific inhibition of individual methylated residues with peptide or small molecule inhibitors will present fewer side effects and will be the most likely successful design of personalised cancer treatment of the future. In line with this, a recent review discussed first-generation inhibitors of arginine methylation currently in pre-clinical or phase I/II clinical trials [214].

## Next steps

While it is clear that protein arginine methylation is emerging as a key regulator of the cell cycle and may offer suitable targets for novel cancer drug development in the future, the immediate research effort should focus on a more detailed and complete cataloguing of PRMTs and their substrates at different stages of the cell cycle and in various cancer types. Hand in hand with this rather mammoth effort would need to be the development of more specific PRMT inhibitors to not only identify which substrate is methylated on which residue by what PRMT, but to also facilitate the study of downstream functions of the methylation and molecular mechanisms of arginine methylation including effects on alternative splicing. It should be noted that these arginine methylation modifications or aberrant PRMT expression levels could differ between cell/tissue type, between cancer types and even between individual patients. Further emphasis should be placed on unravelling the complex interplay and cross regulation of phosphorylation and methylation which has been so far reported in a few proteins but may be a more widespread regulatory mechanism of protein function that may offer further targets of intervention.

## Conclusions

In the coming age of personalised cancer treatment, targeting the specific mutations and anomalous proteins of each patient’s cancer will lead to increased recovery rates. Although our current knowledge of the role of arginine methylation in cell cycle control and cancer development is still in its infancy, it is clear that arginine methylation is an emerging key regulator of the cell cycle that rivals protein phosphorylation in its importance. Further studies are required to determine the exact role that protein arginine methylation plays within the cell cycle, and how this may be used to develop future cancer treatments to target aberrant protein arginine methylation.

## Abbreviations

ABC: ATP-binding cassette
ADMA: asymmetric dimethylarginine
AdOx: adenosine dialdehyde oxidised
AKT/PKB: α serine/threonine-protein kinase/protein kinase B
AMI1: arginine methyltransferase inhibitor 1
APC/C: anaphase-promoting complex/cyclosome
APE1: apurinic/apyrimidinic endonuclease
AR: androgen receptor
ATM: ataxia-telangiectasia mutated
ATR: ataxia-telangiectasia and rad3 related
BER: base excision repair
BRCA: tumor suppressor gene
BUB: budding uninhibited by benzimidazole
C: cysteine
CAK: CDK activating kinase
CBP: CREB-binding protein
CCNE1: cyclin E1 gene
CDC: cell division cycle phosphatase
CDK: cyclin-dependent kinase
CDKI: cyclin-dependent kinase inhibitor
Chk: checkpoint kinase
CIP: CDK interacting proteins
DNA: deoxyribonucleic acid
DP: transcription factor family
DSB: double strand break
E2F: transcription factor family
ER: estrogen receptor
ERCC1: DNA repair endonuclease
FEN1: flap endonuclease 1
G_0_: resting phase of cell cycle
G_1_ phase: first gap phase of cell cycle
G_2_ phase: second gap phase of cell cycle
GADD45: growth arrest and DNA damage-inducible 45α
GAR: glycine–arginine rich
GSK3β: glycogen synthase kinase 3 β
H: histidine
hnRNPUL1: heterogenous nuclear ribonucleo protein
INCENP: inner centromere protein
INK4: inhibitors of CDK4
JMY: junction mediating and regulatory protein
K: lysine
KIP: kinase inhibitory proteins
M phase: mitosis phase of cell cycle
MAD: mitotic-arrest deficient
MCC: mitotic checkpoint complex
MDM4: murine double minute 4
MMA: monomethyl arginine
MMS4: DNA repair endonuclease
MRE11: meiotic recombination 11
MRN: MRE11/Rad51/Nbs1 complex
MUS81: DNA repair endonuclease
NBS1: nibrin
NHEJ: non-homologous end joining
p53: tumor suppressor protein p53
p53BP1: p53-binding protein 1
PCNA: proliferating cell nuclear antigen
PGM: proline glycine methionine motif
PI3K: phosphatidyl inositol-3-kinase
PKC: protein kinase C
pRb: retinoblastoma protein
PRMT: protein arginine methyltransferase
PSA: prostate specific antigen
PTEN: phosphatase and tensin homolog
R: arginine
Rad51: DNA recombination protein
RFC: replication factor C
RNA: ribonucleic acid
RPA: DNA repair protein
S phase: DNA synthesis phase of cell cycle
SAC: spindle assembly checkpoint
SDMA: symmetric dimethylarginine
SF: splicing factor
SR: serine–arginine rich
TFIIH: transcription factor IIH
TRF: telomere repeat binding factor
UBAP2L: ubiquitin-associated protein 2-like
XPA: DNA repair protein
XPC: DNA repair gene
XPF: DNA repair endonuclease
XPG: DNA repair endonuclease
XRCC: DNA base excision repair gene
